# Regularity of Toll-Like Receptors in Bovine Mammary Epithelial Cells Induced by *Mycoplasma bovis*

**DOI:** 10.3389/fvets.2022.846700

**Published:** 2022-04-07

**Authors:** Jinghan Yang, Yuhui Liu, Changjie Lin, Rui Yan, Zhengzhi Li, Qiuhui Chen, Haiyan Zhang, Haojun Xu, Xi Chen, Yingyu Chen, Aizhen Guo, Changmin Hu

**Affiliations:** ^1^College of Veterinary Medicine, Huazhong Agricultural University, Wuhan, China; ^2^State Key Laboratory of Agricultural Microbiology, Huazhong Agricultural University, Wuhan, China

**Keywords:** mastitis, bovine mammary epithelial cells, *M. bovis*, TLRs, pathogen-host interaction, signal pathways

## Abstract

Mastitis is one of the most common and significant infectious diseases in dairy cattle and is responsible for significant financial losses for the dairy industry globally. An important pathogen of bovine mastitis, *Mycoplasma bovis* (*M. bovis*) has a high infection rate, requires a long course of treatment, and is difficult to cure. Bovine mammary epithelial cells (BMECs) are the first line of defense of the mammary gland, and their natural immune system plays a critical role in resisting *M. bovis* infection. This study aimed to explore and demonstrate the regularity of Toll-like receptors (TLRs) activation during *M. bovis* infection and their function during *M. bovis* mastitis. An *in vitro* model of *M. bovis*-induced mastitis showed that the expression of IL-6, IL-8, and TNF-α increased significantly following infection. *M. bovis* infection also upregulated the expression of TLR1/2/6 on the cell membrane and TLR3/9 in the cytoplasm. There is a crosstalk effect between TLR1–TLR2 and TLR2–TLR6. Furthermore, *M. bovis* infection was found to activate the TLR1/2/6/9/MyD88/NF-κB and TLR3/TRIF/IRF signal transduction pathways, which in turn activate inflammatory factors. These findings lay the theoretical foundation for understanding the pathogenesis of *M. bovis*, permitting the development of effective measures for preventing and controlling *M. bovis* mastitis.

## Introduction

Mastitis is a highly prevalent inflammatory disease that ranks first among diseases that affect dairy cows. It causes significant economic losses and severely impacts animal welfare and milk production (1). Bovine mastitis is caused by the co-infection of various pathogenic microorganisms, including bacteria, fungi, *Mycoplasma*, and viruses (2). Mastitis caused by *Mycoplasma bovis* (*M. bovis*) has severely restricted the development of the dairy cow breeding industry because of its long-lasting effects, fast transmission, and poor response to therapy (3).

*Mycoplasmas*, the smallest and most pleomorphic self-replicating prokaryotes, reside either on eukaryotic cell membranes or inside the cells themselves (4). *Mycoplasmas* are complex bacteria, and broad-spectrum antibiotic treatment is not effective against most *Mycoplasmas*. *Mycoplasmas* are pleomorphic due to their lack of cell walls, allowing them to quickly reach and bind to the target surface, and achieve membrane fusion with the host (5). *Mycoplasmas* contain a rich variety of hydrophobic proteins that are specifically soluble in TritonX-114-lipid associated membrane proteins (LAMPs) (6). The pathogenic mechanism of *M. bovis* is caused mainly by the host cells' recognition of LAMPs through their TLRs, triggering an inflammatory response (7). Some *Mycoplasmas* also cause cell damage through their secretions or metabolites. *Mycoplasmas* have developed a variety of strategies to resist and manipulate the host immune system, including evading the host's immune response via surface antigen mutations. Lysnyansky (8) found that *M. bovis* can evade host immunity through variable surface lipoproteins. However, the definitive pathophysiologic mechanisms of *M. bovis* remain unclear.

Breast tissue is protected by both innate and acquired immunity. Innate immunity is dominant during the initial stages of infection, with mammary epithelial cells constituting an essential part of innate immunity. Host cells have a complete set of natural immune systems to resist the invasion of pathogenic microorganisms, called pattern recognition receptors (PRRS). These include TLRs, NOD-like receptors (NLRs), and RIG-I like receptors (RLRs) (9). TLRs are key regulators of both innate immunity and adaptive immunity and are also the best-studied family of PRRs. It has been estimated that most mammalian species have 10 to 15 types of TLRs, and that the distribution of certain TLRs varies between species. TLRs are crucial to the pathogenesis, prevention, and control of mastitis (10–13). Takeda (14) found that TLR2/TLR6 could identify the diacylated lipoprotein M161Ag (MALP404) derived from *Mycoplasma fermentum*. Other studies have shown that *M. bovis* activates the ERK1/2 MAPK pathway through TLR2 and TLR4, promotes the secretion of CXCL8, and intensifies the host's inflammatory response (15). *M. bovis* can also combine with TLR1/2 to stimulate MMP9 production, thereby worsening mastitis (16). TLR recognition mediates the activation of innate immunity to instruct the development of an effective acquired immune response. Thus far, the investigation of Toll-like receptors of bovine mammary epithelial cells induced by *M. bovis* is a breakthrough in the treatment of mastitis.

Here we established a BMECs model of *M. bovis* infection *in vitro* and studied the mechanism of the TLRs signaling pathway after *M. bovis* infection, thereby identifying the types of TLRs and triggered inflammatory signal pathways that are involved in the regulation of *M. bovis* infection. Such associations are key to furthering our understanding of the immune and pathogenic mechanisms of *M. bovis*.

## Materials and Methods

### Cell Preparation and Co-culture With *M. bovis*

Bovine mammary epithelial cells were extracted from the mammary tissue of Holstein cows during their middle lactation period. The cells were routinely maintained in DMEM/F12 medium, supplemented with 10% fetal bovine serum (Sigma, Bedford, MA), 1% penicillin–streptomycin solution (Hyclone Logan, UT), 5 ng/mL EGF (SinoBiological, Beijing, China), and 5 μg/mL insulin and transferrin (Sigma, Bedford, MA). All cells were stored in incubators at 37°C with 5% CO_2_. Three strains of *M. bovis* were used in this study, 39YC (clinical isolates of *M. bovis* from milk), which was identified and stored in our lab; PG45 (standard *M. bovis* strain, ATCC 25523); and HB0801 (*M. bovis* pneumonia clinical isolates from the lungs, CCTCC # M2010040) (17). *M. bovis* and BMECs were co-cultured at a ratio of 1000:1 and 500:1, respectively (18, 19).

### Immunofluorescence

Cytokeratin 18 immunofluorescence was used to identify the isolated BMECs. The BMECs were fixed in 4% paraformaldehyde and blocked with 5% BSA solution. Following incubation with the primary antibody (anti-Cytokeratin 18 antibody, Bioss) overnight at 4°C, BMECs were then incubated with a secondary antibody.

### Polymerase Chain Reaction

Total RNA was isolated using Trizol reagent (Invitrogen, CA), and cDNA was synthesized using a reverse transcription kit (+gDNA wiper) (Vazyme, Nanjing, China). We examined the MUC-1 gene to identify and distinguish BMECs from BMFBs (bovine mammary fibroblasts) (20). Specific primers for the MUC-1 gene are shown below: forward: 5′-AGATCAAGTTCAGGCCAGGAT-3′; reverse: 5′-CCAGGTTTGTATAAGAGAGGTTGC-3′.

### Quantitative Real-Time PCR

The qPCR reaction system included AceQ® qPCR SYBR Green Master Mix 5 μL, upstream primer 0.2 μL, downstream primer 0.2 μL, ROX Reference Dye1 0.2 μL, cDNA (the template obtained by reverse transcription was diluted 10 times to 50 ng/mL) 1 μL, and ddH_2_O 3.4 μL. The qPCR reaction program was 40 cycles at 95°C for 5 min, 95°C for 30 s, 60.5°C for 30 s, and 72°C for 30 s. The primers used for quantitative real-time PCR are shown in Table 1. Each gene's relative expression (cycle threshold) values were normalized based on β-actin expression. H_2_O wells (NTC = no-template controls) were used as negative controls. The induction values of genes mRNA expression were calculated according to the equation: fold change = 2^−ΔΔCt^. All reactions were performed in triplicate on at least 3 biologic replicates. A full description of the experiments that comply with MIQE (the Minimum Information for Publication of Quantitative Real-Time PCR Experiment) can be found in the Supplementary Material.

**Table 1:** Primer sequences of fluorescence quantitative PCR.

**Genes**	**Primers sequence (5^′^-3^′^)**	
IL-8	F:TGAAGCTGCAGTTCTGTCAAG	R:TTCTGCACCCACTTTTCCTTGG
TNF-α	F:TCTTCTCAAGCCTCAAGTAACAAGC	R:CCATGAGGGCATTGGCATAC
IL-6	F:CAGCAGGTCAGTGTTTGTGG	R:CTGGGTTCAATCAGGCGAT
TLR1	F:ACTTGGAATTCCTTCTTCACGA	R:GGAAGACTGAACACATCATGGA
TLR2	F:GGTTTTAAGGCAGAATCGTTTG	R:AAGGCACTGGGTTAAACTGTGT
TLR3	F:GATGTATCACCCTGCAAAGACA	R:TGCATATTCAAACTGCTCTGCT
TLR4	F:TGCTGGCTGCAAAAAGTATG	R:TTACGGCTTTTGTGGAAACC
TLR5	F:CCTCCTGCTCAGCTTCAACTAT	R:TATCTGACTTCCACCCAGGTCT
TLR6	F:CCTTGTTTTTCACCCAAATAGC	R:TAAGGTTGGTCCTCCAGTGAGT
TLR7	F:TCTTGAGGAAAGGGACTGGTTA	R:AAGGGGCTTCTCAAGGAATATC
TLR9	F:CTGACACCTTCAGTCACCTGAG	R:TGGTGGTCTTGGTGATGTAGTC
TLR10	F:ATGGTGCCATTATGAACCCTAC	R:CACATGTCCCTCTGGTGTCTAA
MyD88	F:ACTATCGGCTGAAGTTGTGC	R:TCCTTGCTTTGCAGGTATTC
TRIF	F:GGAGTCGTCCGAGCAGAAA	R:AGGATGATGAATGCCGAGTG
IRF3	F:GCATCCCTTGGAAGCACG	R:CCTCCGCTAAACGCAACAC

### Western Blot

Confluent BMECs were lysed in ice-cold RIPA buffer with 1 mM PMSF (Vazyme, Nanjing, China). Lysate protein concentration was measured using a BCA protein assay kit (Thermo, Waltham, MA). Protein samples were denatured in boiling water for 10 min, and electrophoresed. Protein bands were transferred to a PVDF membrane and blocked using 5% skim milk in a TBST buffer. The bands were combined with primary antibodies (TLR1, Biobyt, 1:1000; TLR2, TLR9, Avivia Systems Biology, 1:1000; TLR3, Novus, 1:500; TLR6 and IκBα, Santa Cruz Biotechnology, 1:1500 and 1:1000; P65, P-P65, p-IκBα, Cell Signaling Technology, 1:1000; p-c-Jun, Bioss, 1:1000) and slowly rocked overnight at 4°C, then incubated with secondary antibodies at room temperature in TBST buffer. Signals were detected using the ECL method.

### Immunoprecipitation

TLR2 and TLR6 protein expression was measured after BMECs were co-cultured with *M. bovis* for 16 h, and TLR2 and TLR1 protein expression was measured after BMECs were infected with *M. bovis* for 20 h. Total cellular protein was extracted using the conventional method, and concentrations were adjusted using PBS (1 mM PMSF) after the protein concentration was measured. Equal 1 mL protein was incubated with 30 μL 50% protein A/G agarose for 1 h, after which the supernatants were collected. The TLR2 primary antibody was added at 4°C overnight, and then incubated with 20 μL protein A/G agarose on a shaker for 5 h at room temperature. Samples were centrifuged and supernatants were collected for Western blot analysis.

### Statistical Analysis

All values were written as the mean ± SD of at least three independent experiments. Statistical analysis was performed using GraphPad Prism 5.0. The *t* tests were used to identify statistically significant differences between the compared groups, and Western blot results were quantified using NIH Image J software (Bethesda, MD). (^*^p < 0.05 = different; ^**^p < 0.01 = significantly different; ^***^p < 0.001 = extremely significantly different).

## Results

### *M. bovis* Induced IL-6, IL-8, and TNF-α mRNA Expression in BMECs

**Figure 1:**
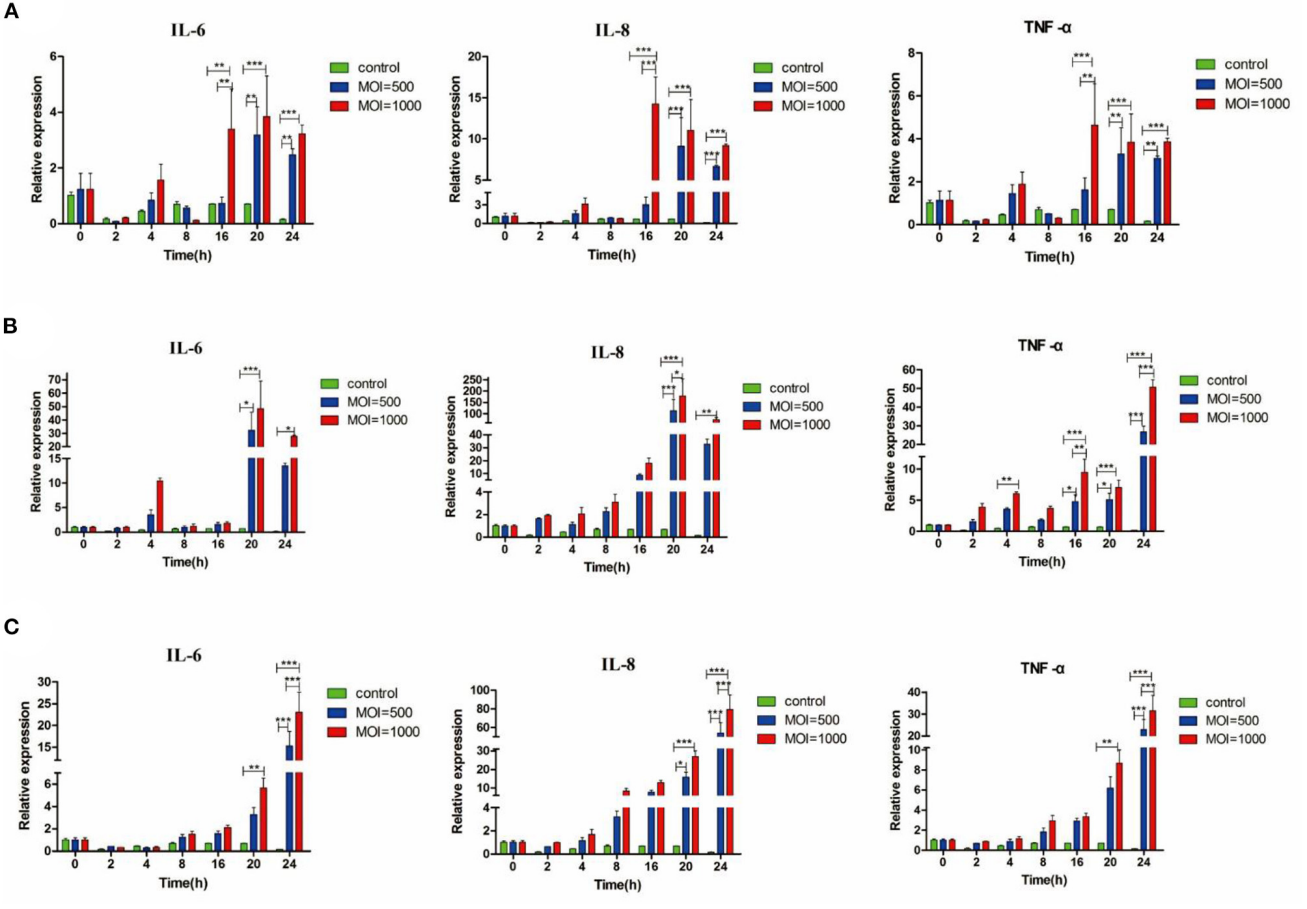
*M. bovis* induced IL-6, IL-8, and TNF-α mRNA expression in BMECs. Relative expression of different cytokines from BMECs infected by three *M. bovis*, respectively, **(A)** BMECs infected by 39YC within 24 h, **(B)** BMECs infected by PG45 within 24 h, **(C)** BMECs infected by HB0801 within 24 h. *p < 0.05, **p < 0.01, and ***p < 0.001.

BMECs were extracted from the mammary tissue of Holstein cows (Supplementary Figure S1). Under MOI conditions of 500 and 1,000, the activity of 39YC, PG45, and HB0801 in BMECs was equivalent with controls, suggesting that BMECs maintained stable activity after being infected with *M. bovis* at MOI = 500 and 1,000 (Supplementary Figure S2). We then measured the expression of inflammatory cytokines such as IL-6, IL-8, and TNF-α in BMECs after infection with *M. bovis* under different strains and at different MOIs (Figures 1A–C). All three *M. bovis* strains induced the expression of inflammatory cytokines by BMECs, and underwent consistent changes based on infection time and MOI. The up-regulated levels of inflammatory factors were more significant at MOI = 1,000 than at MOI = 500. Based on the above experimental results, we established a BMECs model of *M. bovis* infection.

### *M. bovis* Activated TLRs Expression in BMECs

**Figure 2:**
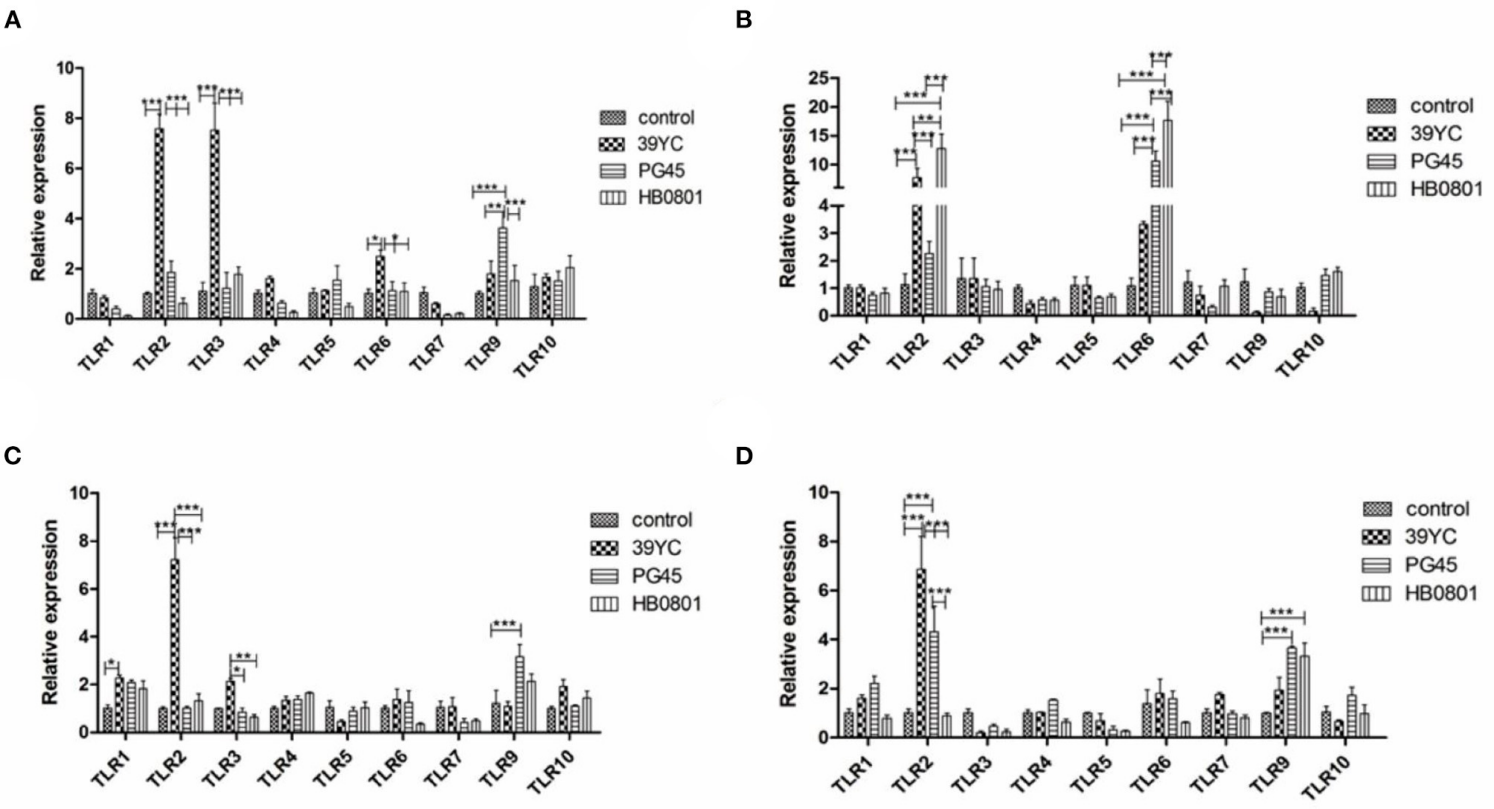
*M. bovis* activated TLRs expression in BMECs. TLRs expression on BMECs induced by *M. bovis* at different time, **(A)** 10 h post-infection (hpi), **(B)** 16 hpi, **(C)** 20 hpi, **(D)** 24 hpi. *p < 0.05, **p < 0.01, and ***p < 0.001.

Cells were harvested for total RNA extraction and the expression of TLR genes 10, 16, 20, and 24 h post-infection (hpi) was measured using PCR. As shown in Figure 2A, the expression of TLR2, TLR3, and TLR6 in the 39YC group increased compared with the control group at 10 hpi. Similarly, the mRNA levels of TLR2 and TLR6 were significantly higher in the 39YC and HB0801 groups than in controls. However, only TLR6 was highly expressed in the PG45 group at 16 hpi (Figure 2B). The mRNA of TLR1 and TLR2 were more highly expressed in the 39YC group than that in the control group, and the mRNA of TLR9 was higher in the PG45 group compared with the control group at 20 hpi (Figure 2C). The expression of TLR2 in the 39YC group was significantly higher than in controls (Figure 2D). There was also a significant difference in TLR2 and TLR9 expression between the PG45 group and the control group, the expression of TLR9 of the HB0801 group was significantly higher than that of the control group at 24 hpi (Figure 2D).

**Figure 3:**
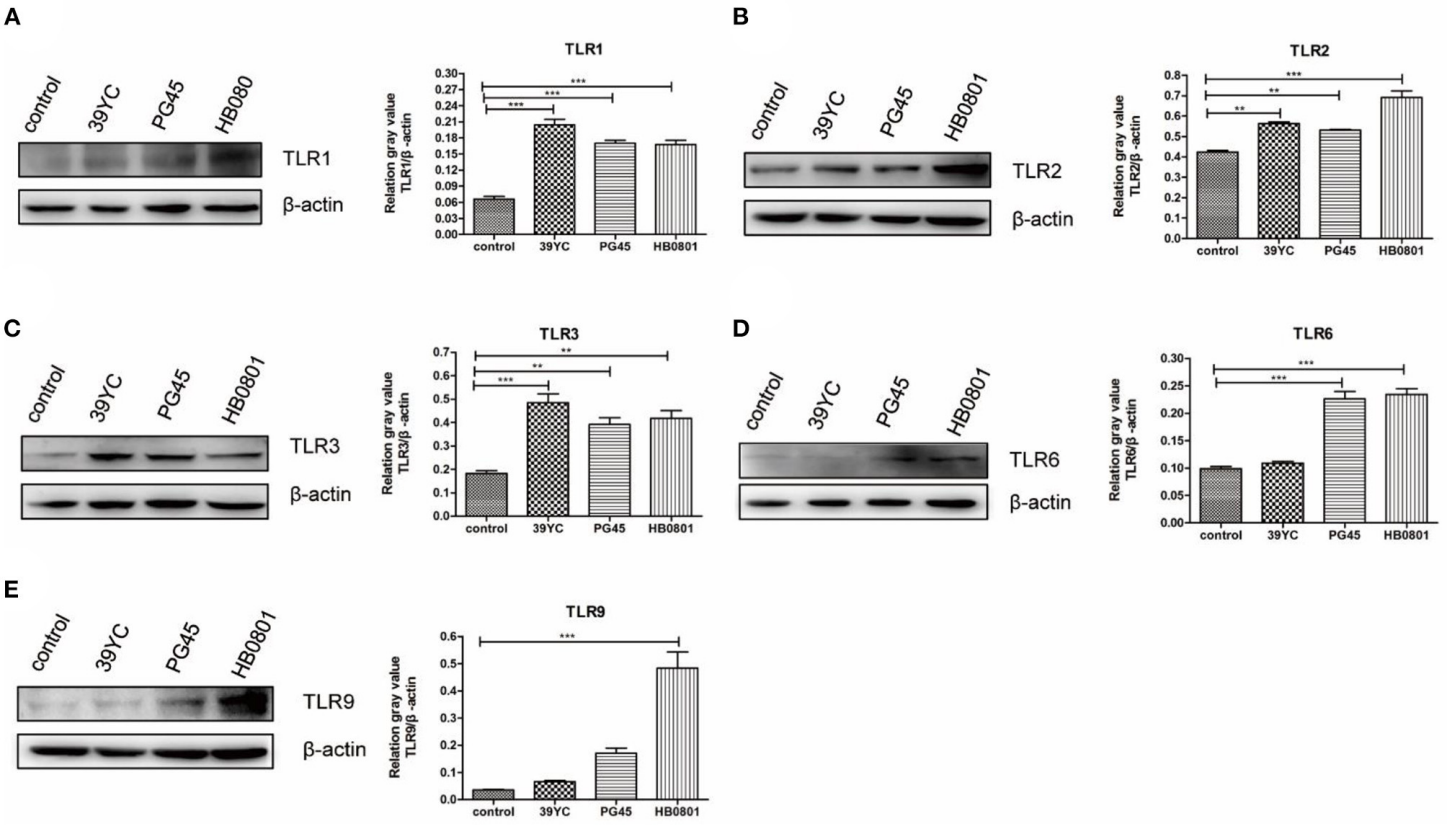
*M. bovis* activated TLRs expression in BMECs. **(A–E)** The protein expression of TLR1, TLR2, TLR3, TLR6, and TLR9 from BMECs infected by *M. bovis*, **(A)** 20 h post-infection (hpi), **(B–D)** 16 hpi, **(E)** 20 hpi. **p < 0.01, and ***p < 0.001.

According to the above results (Figure 2), we found that the expression of TLR2, TLR3, and TLR6 were to increase at 16 hpi, and the expression of TLR1 and TLR9 were increased at 20 hpi. The expression of TLR factors at the indicated times was also detected using Western blot analysis (Figure 3). We found that the expression of TLR2, TLR3, and TLR6 was significantly higher than controls at 16 hpi by the three different stains *M. bovis*. TLR1 and TLR9 protein expression was significantly increased at 20 hpi.

### Roles of TLR1/2/6 in BMECs Induced by *M. bovis*

**Figure 4:**
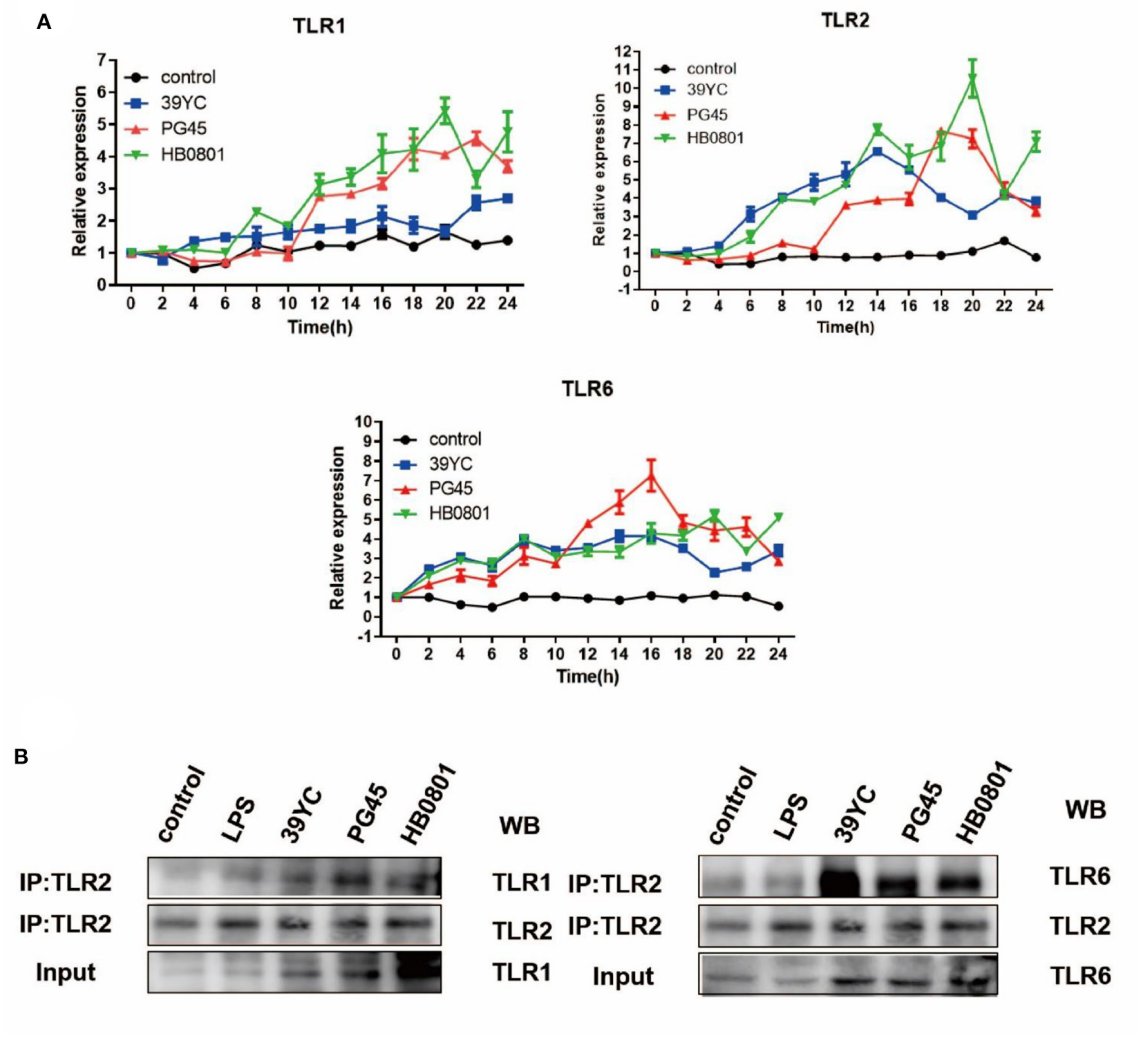
Roles of TLR1/2/6 in BMECs induced by *M. bovis*. **(A)** Time-dependent mRNA expression of TLR1, TLR2, and TLR6 after BMECs infected by *M. bovis*, **(B)** Co-IP for TLR1/TLR2, TLR2/TLR6 after BMECs infected by *M. bovis*.

To detect whether there was a crosstalk between TLR1/TLR2 and TLR2/TLR6, cells were collected at distinct time points after infection for qRT-PCR (Figure 4A). The upregulation of TLR1 began at 4 hpi with 39YC and lasted until 24 hpi. TLR2 began to rise at 4 hpi with 39YC, reaching its peak at 14 hpi. It then downregulated slowly but consistently at an increasing rate. The expression of TLR6 began to rise at 2 hpi with 39YC, and its rate of increase fell 20–22 hpi at a consistent and progressive rate. Similarly, the expression of TLR1 and TLR2 began to rise at 12 hpi with PG45. TLR1 expression fell at a steady increasing rate from 22 to 24 hpi, and TLR2 expression fell at a steady but increasing rate at 18–24 hpi. The expression of TLR6 began to rise at 2 hpi with PG45 and reached its peak at 16 hpi, after which the ascent rate fell until 24 hpi. The expression of TLR1 and TLR2 began to rise at 4 hpi with HB0801 and reached its peak at 20 hpi. TLR6 began to rise at 2 hpi with HB0801, after which it remained upregulated. Similar trends were observed between TLR1/TLR2, and TLR2/TLR6, so immunoprecipitation was used to verify the hypothesis that there was an interaction between TLR1/TLR2 and TLR2/TLR6. This showed that the gray values of TLR1 and TLR6 in the 39YC, PG45, and HB0801 groups were higher than that of the control and LPS groups (Figure 4B), which indicated that TLR1 or TLR6 can combine with TLR2 to recognize the different strains of *M. bovis* after BMECs were infected by *M. bovis*.

### Changes of Inflammatory Signaling Pathways in BMECs Induced by *M. bovis*

**Figure 5:**
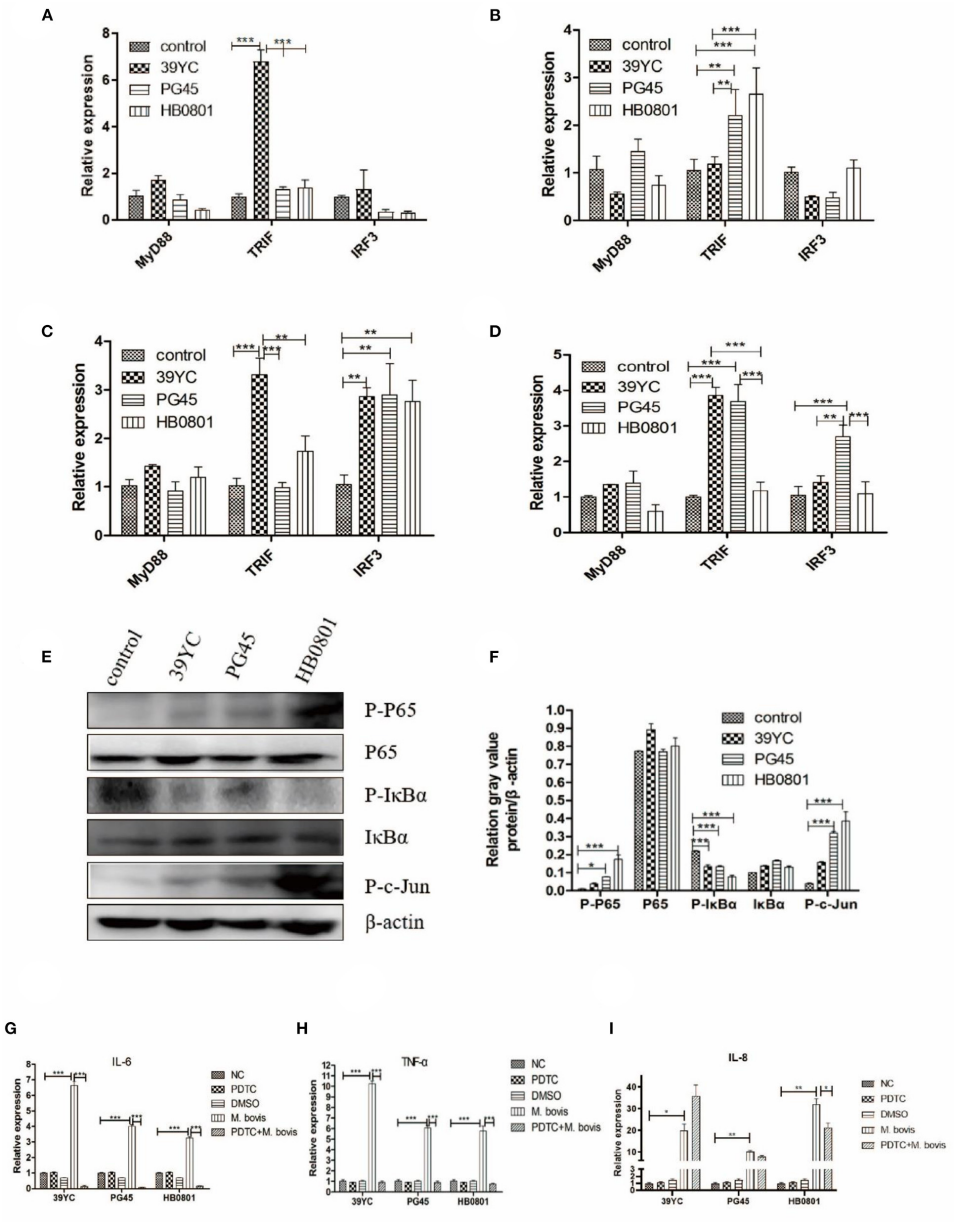
Changes of inflammatory signaling pathways in BMECs induced by *M. bovis*. **(A–D)** Related factors expression of BMECs infected with *M. bovis* at different time points, **(A)** 10 h post-infection (hpi), **(B)** 16 hpi, **(C)** 20 hpi, **(D)** 24 hpi. Proteins expression of TLRs from BMECs infected by *M. bovis*, **(E)** Western Blot, **(F)** gray value analysis. Expression of IL-6, TNF-α, and IL-8 after BMECs infected by *M. bovis* and NF-κB inhibited by PDTC of qRT-PCR, **(G)** IL-6, **(H)** TNF-α, **(I)** IL-8. *p < 0.05, **p < 0 .01, and ***p < 0.001.

To detect changes in the cascade signaling pathway molecules of TLRs after *M. bovis* infection, we surveyed the relative temporal expression of MyD88, TRIF, and IRF3 using qRT-PCR analysis. The expression of TRIF in the 39YC group (Figure 5A) increased significantly at 10 hpi. The expression of TRIF in the PG45 and HB0801 groups increased significantly at 16 hpi (Figure 5B). Compared with the control group, the expression of TRIF in the 39YC group (Figure 5C) increased significantly (*P* < 0.001), and IRF3 expression levels in the 39YC, PG45, and HB0801 groups increased at 20 hpi (*P* < 0.001). The levels of TRIF in the 39YC and PG45 groups, and IRF3 in the PG45 group (Figure 5D) were higher than the control group at 24 hpi (*P* < 0.001). Regarding signaling pathways, the signaling molecules activated downstream of TLRs (NF-κB, TLR4) were analyzed using Western blot after BMECs were co-cultured with *M. bovis* for 20 h (Figure 5E). The results indicated that p-p65 protein levels were markedly increased in the PG45 and HB0801 groups compared with controls (Figure 5F). Moreover, phosphorylation of IκBα protein levels was also obviously reduced in the PG45, HB0801, and 39YC groups than the control group, representing the activation of the NF-κB signaling pathway. In addition, the phosphorylation levels of c-jun were increased in three groups, especially in the PG45 and HB0801 groups. To further investigate the role of the NF-κB pathway in mastitis, we used PDTC to treat with BMECs, an NF-κB inhibitor. The expression of IL-6 and TNF-α was markedly decreased after treatment with PDTC (Figures 5G,H). However, the expression of IL-8 was decreased only in the HB0801 group after treatment with PDTC (Figure 5I).

## Discussion

As a functional part of mammary immunity, BMECs can present certain immunogenic pathogens after infection (21, 22). Our experiment showed that three strains of *M. bovis* did not impair the viability of BMECs within 24 h of infection at MOIs of 500 and 1,000. Beyond that, the changes of inflammatory factors at different time points also found that the expression of IL-6, TNF-α, and IL-8 was increased in BMECs after *M. bovis* infection and showed a change dependent on infection time and MOIs. This confirms the establishment of a stable *in vitro* BMECs inflammation model of *M. bovis*.

The expression of IL-6, a major immune and inflammatory mediator, and the IL-6 level in our experiment began to increase at 4 h post-infection (hpi). While the expression level of IL-6 was increased at 24 hpi, and it participated in the post-infection reaction, there was no increase in the early stage in Liu's study (23). This might be due to the diverse types and culture methods of both the *M. bovis* and BMECs cell lines, which can lead to differences in the virulence of *M. bovis* and the characteristics of the BMECs. TNF-α not only promoted the release of IL-8 (24), but also had the ability to activate NF-κB (25). As TNF-α is a known key inflammatory factor, and the increase of TNF-α expression following exposure to all three *M. bovis* strains confirms their pro-inflammatory effects on BMECs. IL-8 is a chemokine that recruits neutrophils to swarm to sites of infection with multiple pathogens (26). IL-8 secretion increased after *M. bovis* infection (27). All three *M. bovis* strains promoted IL-8 overexpression in the present work, an observation consistent with the findings of Zbinden et al. (28) in their own study of the BMEC immune response to *M. bovis* infection.

TLRs are pattern recognition protein receptors, and they are expressed by many immune and epithelial cells (29, 30). TLR1-10 can be expressed by bovine tissues, but the expression and distribution of TLRs differs between different tissues and cells (31). We showed here that the expression levels of TLR1, TLR2, and TLR6 increased after BMECs were infected with *M. bovis*. It is well-known that TLR1, TLR2, and TLR6 can recognize *Mycoplasma* to initiate innate immunity. The expression levels of TLR3 and TLR9 increased in this study, indicating that *M. bovis* contained some components that could be recognized by TLR3 and TLR9.

To identify the roles of TLR1/2, and TLR2/6 in BMECs in response to *M. bovis*, we measured the changes in TLRs expression at different time points after *M. bovis* infection. TLR2 forms dimers with TLR1 and TLR6 to resist the invasion of *Mycoplasma* (32). The results of the co-immunoprecipitation experiment showed TLR1 and TLR6 were better able to bind to a TLR2 primary antibody following infection with three strains of *M. bovis* compared with controls, which indicated that there was a crosstalk between TLR1/TLR2 and TLR2/TLR6. However, it remains to be further demonstrated whether dimers are formed between TLR-TLR2 and TLR2-TLR6.

Inflammatory signaling pathways are crucial to the pathogenesis of mastitis. Our experiment showed that the expression of both TRIF and downstream IRF3 was increased. The phosphorylation of IκBα protein decreased in the present work, indicating that the NF-κB signaling pathway was activated. JNK is an important member of the MAPK signaling pathway, which requires the phosphorylation of JNK to be activated. This study found that the expression of P-c-Jun protein increased after *M. bovis* infection, indicating that the activation of TLRs promoted MAPK activation. PDTC can inhibit the activation of NF-κB by inhibiting the phosphorylation process (33–36). Cell viability was not affected by 50 μM PDTC in the present work. The expression of IL-6 and TNF-α increased after BMECs were infected with *M. bovis*, then decreased after PDTC intervention, indicating that the expression of these inflammatory factors was associated with the NF-κB. It also indirectly explained the role of TLRs. However, the expression of IL-8 was decreased only in the HB0801 group after treatment with PDTC, increasing in the 39YC group and remaining equivocal in the PG45 group than controls. The different expression trends of IL-8 between *M. bovis* strains may represent differences in their pathogenic mechanisms. The reason for the increased IL-8 level in the 39YC group after PDTC treatment may be that *M. bovis* can regulate the secretion of IL-8 through multiple pathways (37). Other signaling pathways may also be triggered by 39YC, which dominates the regulation of IL-8 in BMECs alongside NF-κB. The specific mechanisms underlying these effects require further study. This study did have some limitations. For instance, which TLRs are related to breast lactation function and inflammation still needs to be explored in future experiments. The innate immunity NLRs caused by *M. bovis* also require further study.

## Conclusion

**Figure 6:**
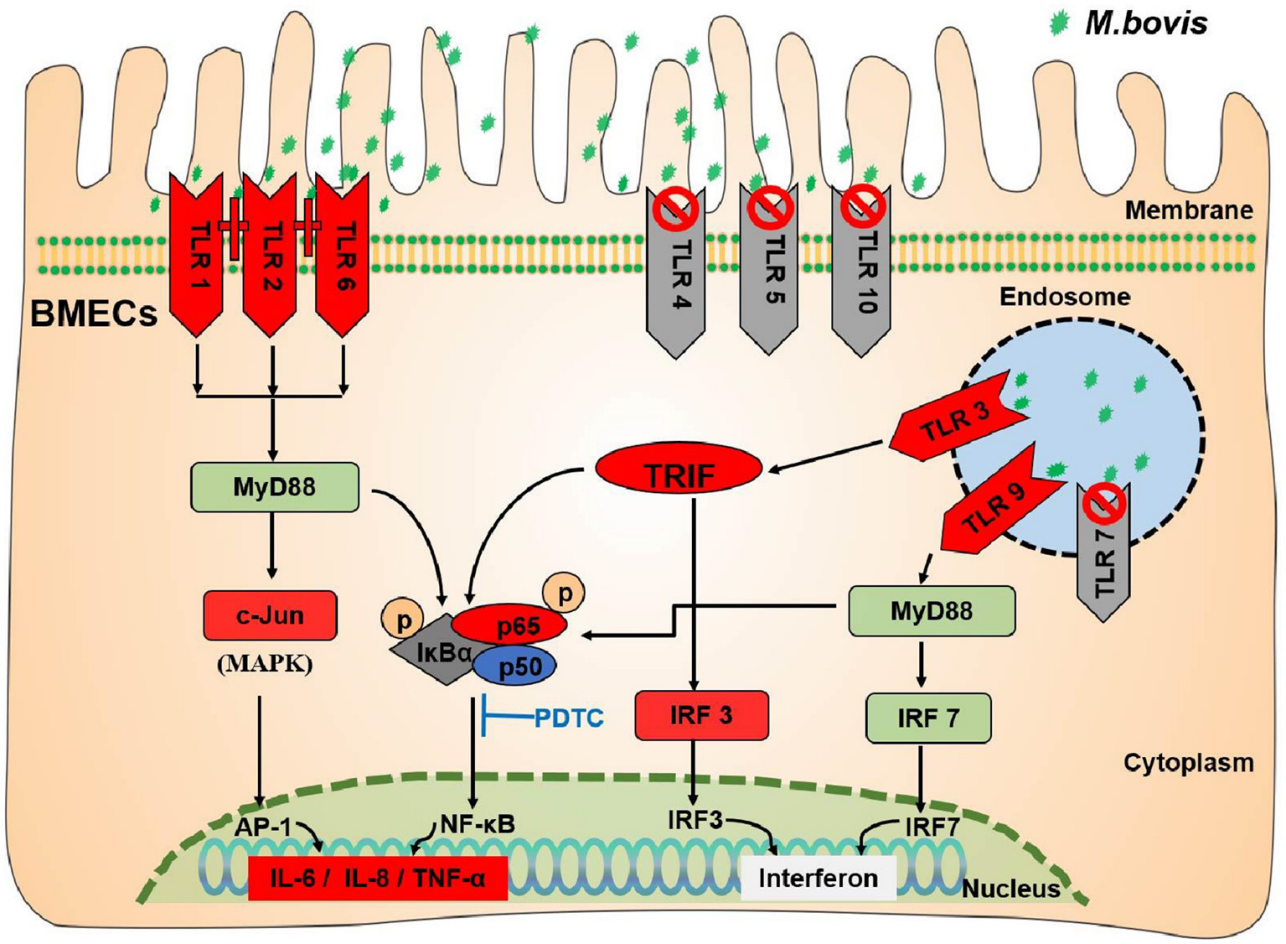
TLRs signaling pathway induced by *M. bovis* infected BMECs. The green stars indicate *M. bovis*. The red (TLR1/2/6/3/9; TRIF/IRF3/c-Jun/P-P65; IL-6/IL-8/TNF-α) in the figure indicates that the expression level increased. The gray (TLR4/5/10/7) indicates that they have no effect during *M. bovis* infection. The dark gray (P-IκBα) indicates that expression decreased during *M. bovis* infection.

This study clearly shows that *M. bovis* infection initiates the innate immune-TLRs signaling pathway of BMECs (Figure 6). *M. bovis* activated TLR1, TLR2, and TLR6 on the BMECs membrane surface and TLR3 and TLR9 pattern recognition receptors in their cytoplasm. TLR2 could combine with TLR1 or TLR6 to initiate immunity. The infectious signal was transmitted downward through the action of the adaptor proteins MyD88 and TRIF, activating the inflammatory signal pathways of NF-κB and MAPK, and increasing the expression of IL-6, TNF-α, IL-8, and interferon IRF3.

## Data Availability Statement

The original contributions presented in the study are included in the article/Supplementary Material, further inquiries can be directed to the corresponding author.

## Author Contributions

JY and CH wrote the manuscript. YL performed the literature retrieval, designed and conducted experiments, and analyzed data. CL, RY, ZL, HX, QC, and HZ performed the literature retrieval. XC, YC, AG, and CH designed the experiments and revised the manuscript. All authors contributed to the article and approved the submitted version.

## Funding

This research was funded by the National Natural Science Foundation of China (Nos. 31972758 and 31101874), the special fund for the China Agriculture Research System (Beef/Yak cattle) (No. CARS-37), Key R&D Program of Hubei Province of China (No. 2020BBA055), and the Fundamental Research Funds for the Central Universities (No. 2662020DKPY014).

## Conflict of Interest

The authors declare that the research was conducted in the absence of any commercial or financial relationships that could be construed as a potential conflict of interest.

## Publisher's Note

All claims expressed in this article are solely those of the authors and do not necessarily represent those of their affiliated organizations, or those of the publisher, the editors and the reviewers. Any product that may be evaluated in this article, or claim that may be made by its manufacturer, is not guaranteed or endorsed by the publisher.
