# Supplementary material for: Regularity of Toll-Like Receptors in Bovine Mammary Epithelial Cells Induced by Mycoplasma bovis
Source: Front Vet Sci. 2022 Apr 7;9:846700. doi: 10.3389/fvets.2022.846700 (PMC9021453; doi:10.3389/fvets.2022.846700)
Supplement: Supplementary file 1 [file Data_Sheet_1.DOCX]

Regularity of Toll-like receptors in bovine mammary epithelial cells induced by *Mycoplasma bovis*

Supplementary Materials

(Supplementary Figures)


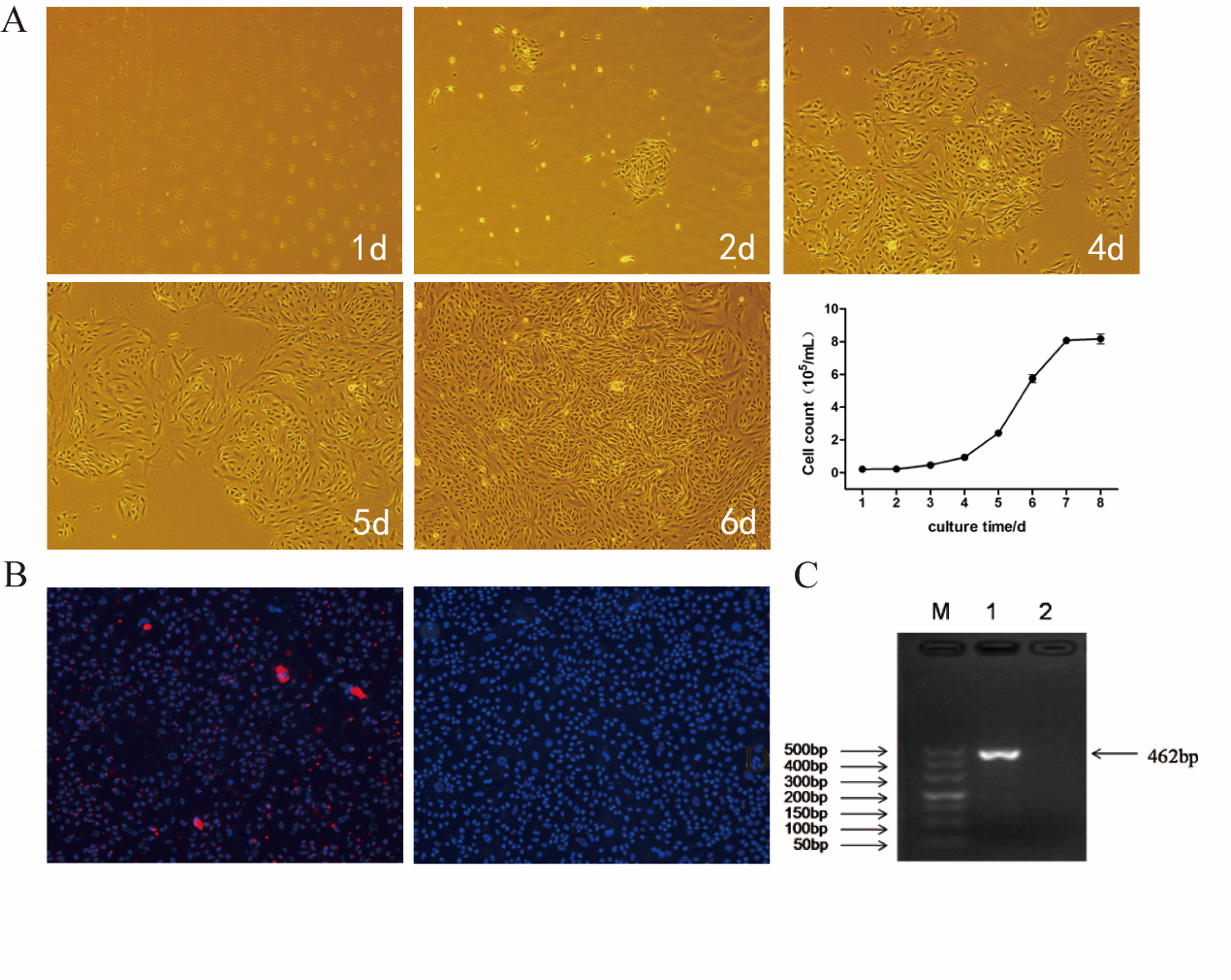


**Supplementary Figure 1.** **The isolation and identification of BMECs**

(A): Observation of BMECs morphology at different culture time (d) and Growth Culture of primary BMECs; (B): Fluorescent image of BMECs stained for cytokeratin18 (Red), negative control of bovine mammary fibroblasts; (C): Identification of BMECs, MUC-1 PCR amplification. M: Marker, 1: BMECs, 2: fibroblasts.


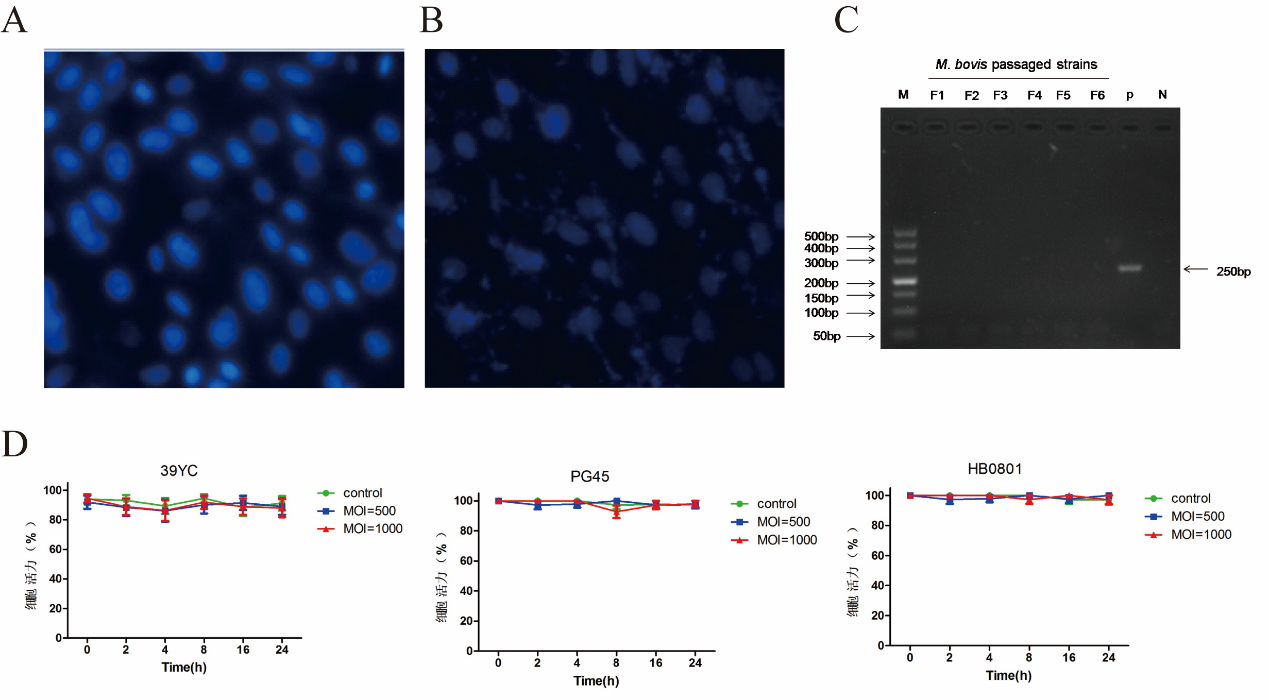


**Supplementary Figure 2.** **Co-culture of M. bovis and BMECs**

Trypan blue cell viability test for BMECs infected by *M. bovis*.


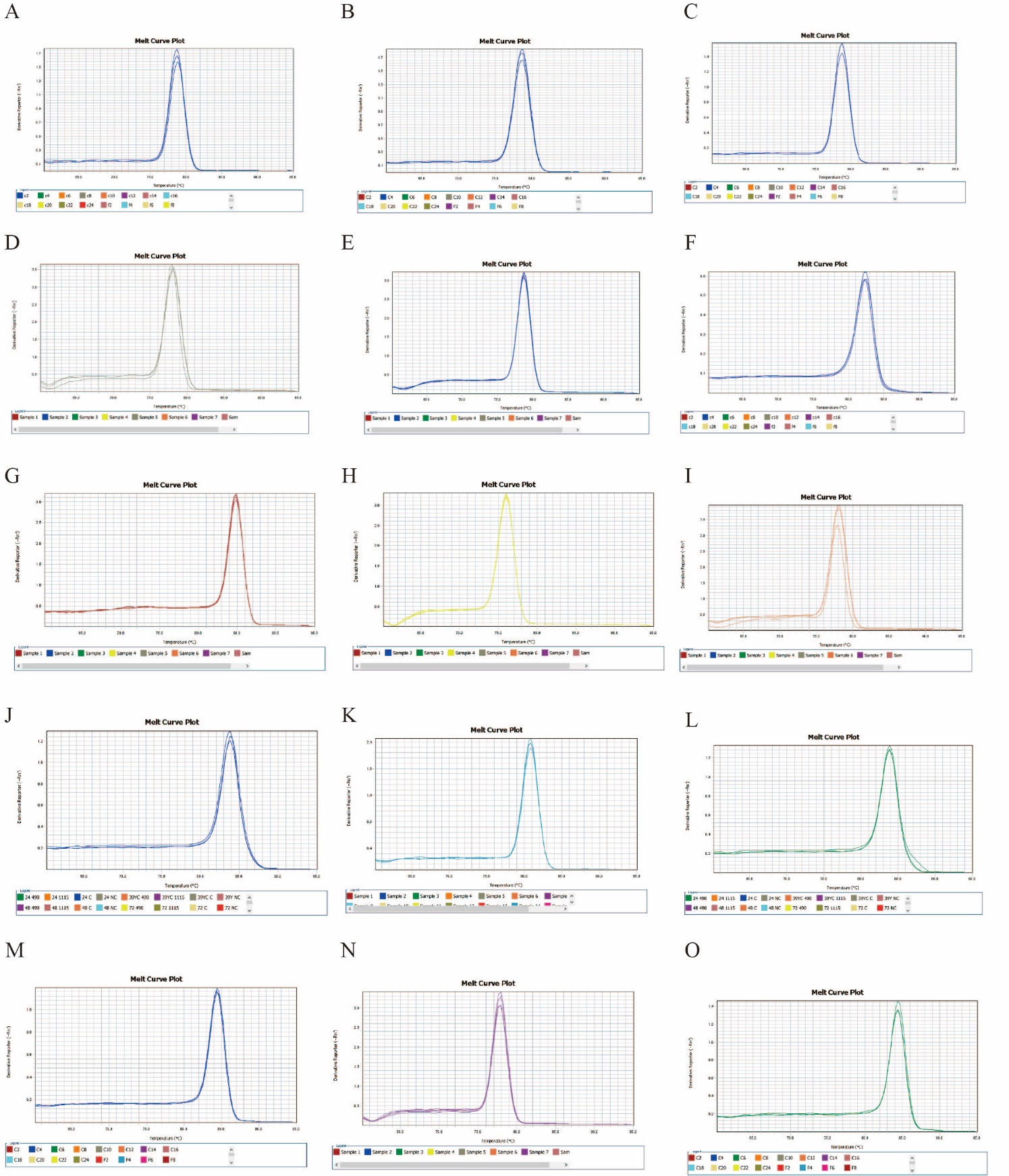


**Supplementary Figure 3 The melting curve of primers**

(A)-(F):TLR1-TLR6; (G)-(I):TLR7, TLR9, TLR10; (J)-(L): IL-6, IL-8, TNF-α;

(M)-(O):MYD88,IRF3,TRIF
