# Supplementary material for: Regularity of Toll-Like Receptors in Bovine Mammary Epithelial Cells Induced by Mycoplasma bovis
Source: Front Vet Sci. 2022 Apr 7;9:846700. doi: 10.3389/fvets.2022.846700 (PMC9021453; doi:10.3389/fvets.2022.846700)
Supplement: Supplementary file 2 [file Data_Sheet_2.DOCX]

Regularity of Toll-like receptors in bovine mammary epithelial cells induced by *Mycoplasma bovis*

Supplementary Materials

(Supplementary Text 1)

**A full description of the experiment that comply with MIQE**

1. **EXPERIMENTAL DESIGN**

- **(A)Definition of experimental and control groups.**

**Response** a randomly selected subset of subjects.

**(B)Number within each group.**

**Response** three**.**

1. **Sample**

- **(A)Description.**

**Response** the experiments were performed in strict accordance with the requirements of biosafety rules, and no contamination was found during the experiments.

**(B)Sample storage conditions and duration (especially for FFPE samples).**

**Response** sample was placed in cryopreserve and stored at a −80 ℃ for 1 year.

1. **NUCLEIC ACID EXTRACTION**

**(A)Source of additional reagents used.**

**Response** trizol (Invitrogen, CA, USA), chloroform, isopropanol, ethanol were of analytical grade and were purchased from Shanghai Chemical Reagent Company.

**(D)Nucleic acid quantification: instrument and method, purity (A260/A280)**

**Response** A260/A280 ratio ≥1.8.

1. **REVERSE TRANSCRIPTION**
2. **Complete reaction conditions: amount of RNA and reaction volume.**

**Response** the template cDNA obtained by reverse transcription was 500ng/ml, and the total reaction volume was 10μL.

1. **Complete reaction conditions: temperature and time.**

**Response** 42℃, 2min; 50℃, 15min; 85℃, 5sec.

1. **Complete reaction conditions: manufacturer of reagents and catalogue numbers.**

**Response** Hiscript® Ⅱ Q RT SuperMix for qPCR(+gDNA wiper) (Vazyme, Nanjing, China).

1. **Storage conditions of cDNA**

**Response** cDNA was placed in cryopreserve and stored at a −20 ℃.

1. **qPCR OLIGONUCLEOTIDES**
2. **Manufacturer of oligonucleotides.**

**Response** the primers for quantitative real-time PCR are synthesized by Tsingke Biological Technology Company (Beijing, China).

1. **qPCR PROTOCOL**
2. **Complete reaction conditions:** **reaction volume and amount of cDNA/DNA.**

**Response** AceQ® qPCR SYBR® Green Master Mix 5 μL, upstream primer 0.2 μL, downstream primer 0.2 μL, ROX Reference Dye1 0.2 μL, cDNA (the template obtained by reverse transcription was diluted 10 times to 50ng/ml) 1 μL, and ddH_2_O 3.4 μL.

1. **Complete reaction conditions:** **buffer/kit identity and manufacturer.**

**Response** AceQ® qPCR SYBR® Green Master Mix (Vazyme, Nanjing, China).

**(C)Manufacturer of plates/tubes and catalog number.**

**Response** 384-well plates were from ABI Company.

**(D)** **Complete thermocycling parameters.**

**Response** the qPCR reaction program was 40 cycles of 95°C for 5 min, 95°C for 30s, 60.5°C for 30s, and 72°C for 30s.

1. **Manufacturer of qPCR instrument.**

**Response** the ViiA7 real-time PCR instrument were from ABI Company.

1. **DATA ANALYSIS**
2. **Description of normalisation method.**

**Response** CT values were normalized using 2^−ΔΔCT^ methods.

1. **Number and concordance of biological replicates.**

**Response** all reactions were done in triplicate and on at least 3 biological replicates, with consistent results.

1. **Number and stage (RT or qPCR) of technical replicates**

**Response** experiments were repeated three times with 3 technical replicates in each assay.

1. **Statistical methods for result significance.**

**Response** the t test was used to determine the statistical significance of the differences between compared groups, (* P < 0.05, differences; ** P < 0.01, significant differences; ***P < 0.001, extremely significant).

**(E)Software (source, version)**

**Response** GraphPad Prism 5.0 (GraphPad Software Inc., San Diego, CA).

1. **Primer BLAST results**

- Specificity of the selected primers was tested using NCBI’s Primer-BLAST (https://www.ncbi.nlm.nih.gov/tools/primer-blast/). In “Nucleotide Blast” we entered forward primer and reverse primer. Because the specificity checking only works with primer pairs, not with single oligonucleotides the test was run three times. The results were similar with expected results (Table 1). In our experiment, we obtained a signal from the specific reporting dye only (Figure 1).

**Table 1 the primers for qPCR**

| Genes | Primers | Sequences(5’to 3’) | Size  (bp) | Reference |
| --- | --- | --- | --- | --- |
| IL-6 | forward | CAGCAGGTCAGTGTTTGTGG | 205 | EU276071 |
|  | reverse | CTGGGTTCAATCAGGCGAT |  |  |
| IL-8 | forward | TGAAGCTGCAGTTCTGTCAAG | 202 | S82598 |
|  | reverse | TTCTGCACCCACTTTTCCTTGG |  |  |
| TNF-α | forward | TCTTCTCAAGCCTCAAGTAACAAGC | 104 | EU276079 |
|  | reverse | CCATGAGGGCATTGGCATAC |  |  |
| TLR1 | forward | ACTTGGAATTCCTTCTTCACGA | 176 | NM_001046504 |
|  | reverse | GGAAGACTGAACACATCATGGA |  |  |
| TLR2 | forward | GGTTTTAAGGCAGAATCGTTTG | 190 | NM_174197 |
|  | reverse | AAGGCACTGGGTTAAACTGTGT |  |  |
| TLR3 | forward | GATGTATCACCCTGCAAAGACA | 195 | NM_001008664 |
|  | reverse | TGCATATTCAAACTGCTCTGCT |  |  |
| TLR4 | forward | TGCTGGCTGCAAAAAGTATG | 213 | NM_174198 |
|  | reverse | TTACGGCTTTTGTGGAAACC |  |  |
| TLR5 | forward | CCTCCTGCTCAGCTTCAACTAT | 172 | AY634631 |
|  | reverse | TATCTGACTTCCACCCAGGTCT |  |  |
| TLR6 | forward | CCTTGTTTTTCACCCAAATAGC | 154 | NM_001001159 |
|  | reverse | TAAGGTTGGTCCTCCAGTGAGT |  |  |
| TLR7 | forward | TCTTGAGGAAAGGGACTGGTTA | 205 | AY487802 |
|  | reverse | AAGGGGCTTCTCAAGGAATATC |  |  |
| TLR9 | forward | CTGACACCTTCAGTCACCTGAG | 156 | NM_183081 |
|  | reverse | TGGTGGTCTTGGTGATGTAGTC |  |  |
| TLR10 | forward | ATGGTGCCATTATGAACCCTAC | 248 | NM_001076918 |
|  | reverse | CACATGTCCCTCTGGTGTCTAA |  |  |
| MyD88 | forward | ACTATCGGCTGAAGTTGTGC | 138 | NM_001014382 |
|  | reverse | TCCTTGCTTTGCAGGTATTC |  |  |
| TRIF | forward | GGAGTCGTCCGAGCAGAAA | 201 | NM_001030301 |
|  | reverse | AGGATGATGAATGCCGAGTG |  |  |
| IRF3 | forward | GCATCCCTTGGAAGCACG | 180 | BC102119 |
|  | reverse | CCTCCGCTAAACGCAACAC |  |  |


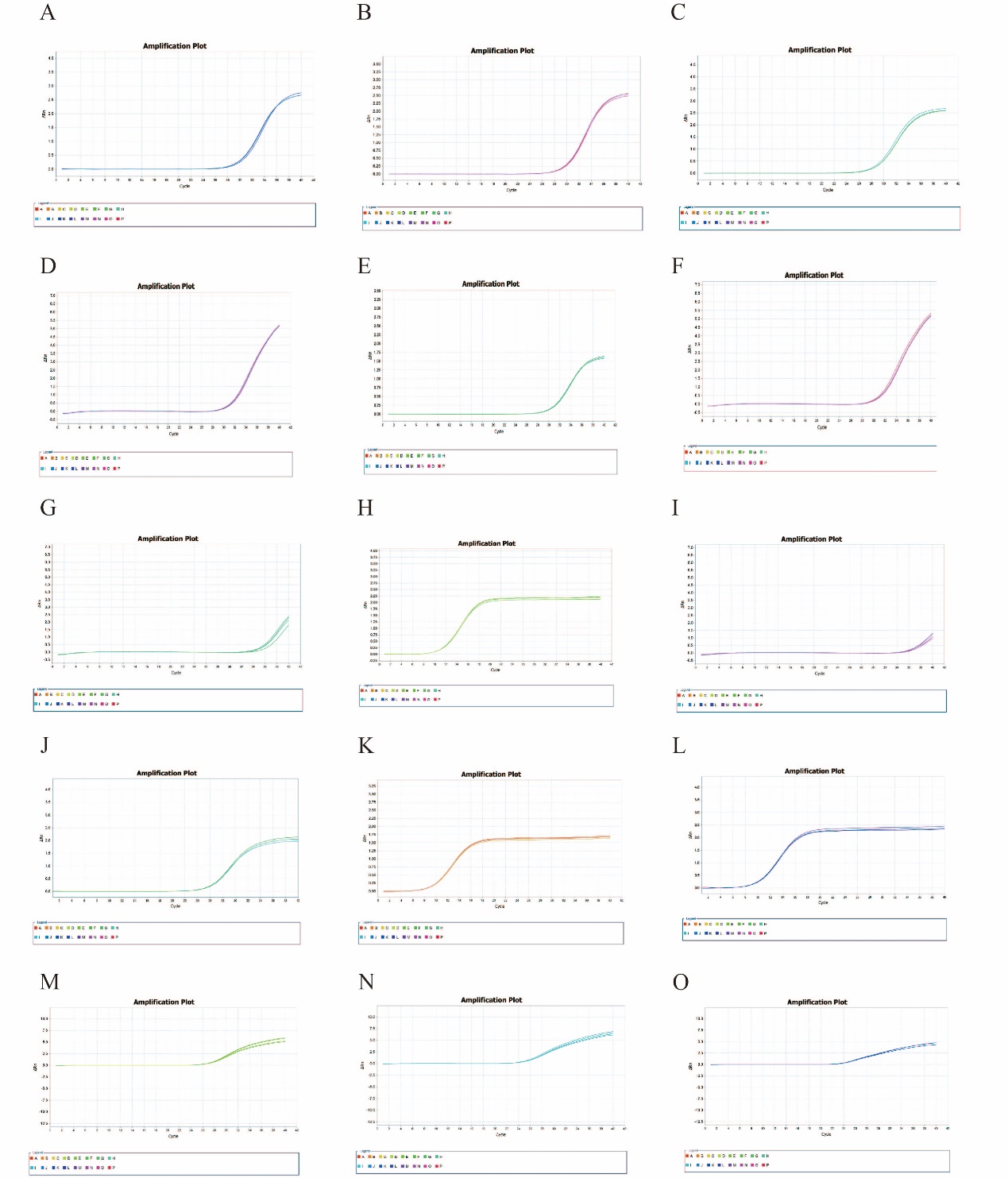


**Figure 1 the amplification curve of primers**

(A)-(F):TLR1-TLR6,(G)-(I):TLR7, TLR9, TLR10,(J)-(L):IL-6,IL-8,TNF-α,(M)-(O):MYD88,IRF3,TRIF.
