# Supplementary material for: Regularity of Toll-Like Receptors in Bovine Mammary Epithelial Cells Induced by Mycoplasma bovis
Source: Front Vet Sci. 2022 Apr 7;9:846700. doi: 10.3389/fvets.2022.846700 (PMC9021453; doi:10.3389/fvets.2022.846700)
Supplement: Supplementary file 3 [file Data_Sheet_3.DOCX]

**Identification of *Mycoplasmas bovis* 39YC**


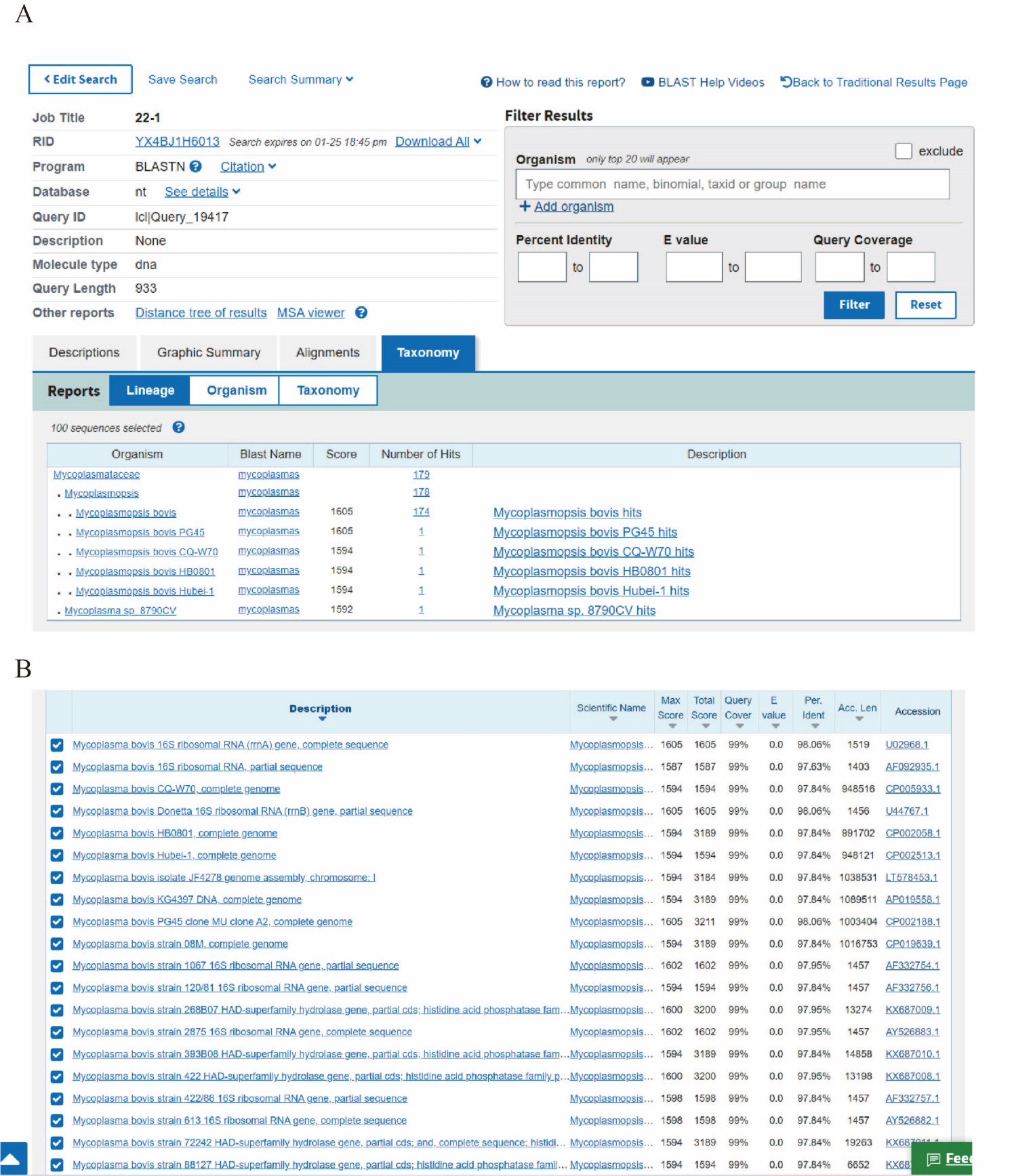


**Identification of *Mycoplasmas bovis* 39YC**(A) The sequence of 39YC homology. (B) The sequence similarity of 39YC with *M.bovis*.
